# Surface Topography Description of Threads Made with Turning on Inconel 718 Shafts

**DOI:** 10.3390/ma16010080

**Published:** 2022-12-22

**Authors:** Bartłomiej Krawczyk, Piotr Szablewski, Michał Mendak, Bartosz Gapiński, Krzysztof Smak, Stanisław Legutko, Michał Wieczorowski, Edward Miko

**Affiliations:** 1 Faculty of Mechanical Engineering, Poznan University of Technology, Piotrowo 3, 60-965 Poznan, Poland; 2 Pratt & Whitney Kalisz, Elektryczna 4a, 62-800 Kalisz, Poland; 3 Institute of Gears Research Excellence Center, The President Stanislaw Wojciechowski Calisia Univeristy, Nowy Świat 4, 62-800 Kalisz, Poland; 4 ITA, Poznańska 104, Skórzewo, 60-185 Poznan, Poland; 5 Faculty of Mechatronics and Mechanical Engineering, Kielce University of Technology, Tysiąclecia Państwa Polskiego 7, 25-314 Kielce, Poland

**Keywords:** thread, turning, topography, Inconel 718

## Abstract

The technology of producing threads, especially in materials that are difficult to cut, is a rare subject of research and scientific publications. The requirements for the production of these elements apply not only to the geometry, but also to the quality of the surface obtained. This is particularly important in the aviation industry, where the durability of the threaded connection affects passenger safety. Due to the design of the thread, the quality of its surface is assessed visually in industrial practice. The authors of this study decided to examine the surface topography of external threads made by turning on Inconel 718 shafts in order to confirm the visual evaluation, as well as to investigate the influence of such factors as cutting speed, turning direction and type of profile. Three types of contours were cut for the research: triangular, trapezoidal symmetrical and trapezoidal asymmetrical. Turning of each was carried out twice at cutting speeds *v_c_* = 17 m/min and *v_c_* = 30 m/min. On each of the threads, the side surface of the profile made in the direction of the insert feed and the opposite surface were examined. The surface texture parameters *Sa, Sq, Sp, Sv, Sz, Ssk* and *Sku* were determined and compared. It was noticed that the thread surfaces show a tendency to irregular roughness, which was confirmed by the analysis of the *Sku* and *Ssk* coefficients. The sides of the contours made in the direction of the insert feed are characterized by a higher roughness in relation to the opposite sides, which may result from high cutting forces and difficulties with chip evacuation. With the cutting speed being considered, lower values of *Sa* and *Sq* were obtained for *v_c_* = 17 m/min, which differed from the visual assessment, proving its high subjectivity.

## 1. Introduction

Optimisation of the production processes is an important feature of the present-day industry. Increasing costs of labour cause enterprises to aim at reduction of the product manufacturing time. However, it has to be kept in mind that these actions are to be performed maintaining the determined level of the product quality. Increasing productivity by changing cutting parameters or by the use of a greater number of modern cutting tools certainly influences the obtained surface quality [1,2]. It is especially important in respect to the safety of exploitation of the parts produced, e.g., in the aircraft industry, where the roughness requirement of the side of a loaded BUTTRESS thread is *Ra* = 0.8 µm. In order to meet the set requirements, precise checking of the manufactured surfaces is necessary [3]. In most cases, analysis of the roughness profile is sufficient, but it does not provide full information about the surface irregularity [4]. In the case of contact of two surfaces, examination of just the 2D profile often neglects the most significant area of the occurrence of the real contact [5]. Further detail, e.g., concerning local irregularities, can be obtained by three-dimensional projection of the surface.

Researchers are increasingly taking advantage of the possibility to use topography when analysing the phenomena occurring in the process of machining. Krolczyk et al. have proved the advantageous influence of coolant on the quality of the surface of turned stainless steel by means of 3D analysis [6,7]. Wojciechowski et al. have proven the positive influence of the inclination of the spherical milling cutter on the topography of hardened steel [8]. Liang et al. have assessed alterations on the surface of titanium alloy with the wear of the cutting tool [9]. Zawada-Tomkiewicz has compared the quality of the hardened steel after turning with an uncoated tool to that of the steel turned with a coated tool [10]. Nieslony et al., in one of their papers, have observed the influence of vibration on the surface irregularities in the case of exceeding the stable range of turning parameters [11]. In another work, researchers examined the area of contact of steel and titanium when drilling explosively welded bimetal [12,13]. Although the researchers of the above-mentioned works studied and demonstrated the influence of a number of factors, such as cutting parameters, machining strategy, the influence of coolant or tool coating on the obtained topography, none of these works focuses on thread machining. At this point, it is worth emphasizing the novelty of this work and the effort we undertook to examine the hard-to-reach side surface of the threads.

Obtaining of a smooth surface without defects is a particularly important task when forming hard-to-machine aircraft materials, such as Inconel 718, which has a lower machinability than do conventionally used alloys, e.g., steel [14,15]. The cutting tool during the turning of this superalloy wears quickly, mainly due to the high temperature that is absorbed by the insert. The Inconel structure contains titanium and niobium oxide inclusions, which have a huge impact on increased abrasion [16]. This heat-resistant alloy, built on the basis of nickel, is, among others, susceptible to forming a kind of sticking when turned without coolant [17]. The necessity of leading heat out of the cutting zone and of the selection of the cutting speed significantly influences the tool wear and the quality of the obtained surface [18,19]. For this reason, a lower cutting speed must be used, and the use of cooling is extremely important compared to conventional alloys. De Souza et al. have performed analysis of the surface of Inconel after grinding [20]. Przestacki and Chwalczuk, using an analysis of three-dimensional imaging, have determined the parameters allowing for obtaining good surface quality in turning another nickel matrix alloy—Waspaloy [21]. Lu et al. have elaborated a mathematical model for the prediction of surface topography after the micromilling of Inconel [22]. Although scientists are increasingly researching superalloys, the area is still understudied. A specific case consists of threads made with these kind of materials, the turning of which is one of the most challenging processes of machining. In order to obtain satisfying surface quality, it is necessary to ensure optimum cutting speed and depth in the individual passes, considering the large resistance forces resulting from the large length of the cutting edge. Particular care should be taken when turning thin-walled elements characterized by a tendency toward deformation and vibration. When surveying literature, one can identify a gap in the state of knowledge concerning the threads in Inconel 718 and the measurements of its surfaces [23]. Nevertheless, researchers are still seeking a solution aimed at the improvement of thread surfaces in superalloys. For instance, He et al. reported the positive influence of ultrasonic vibration-assisted machining on the obtained morphology of the surfaces of threads turned in Inconel 718 [24].

We thus aimed to examine the 3D topography of the threads turned in the above-mentioned material, considering such factors as cutting speed or the direction of turning. It should be pointed out here that the cutting speed is of key importance here due to the fact that the value of the feed in thread turning is constant and determined by its pitch. Various types of threads were tested with particular types differing primarily in the angles of the flanks, which were found to have a significant impact on the distribution of cutting forces during turning. With large BUTTRESS thread angles, i.e., 45°, there are large forces in the radial direction, which may result in an increased tendency to vibration, especially in low-rigid elements. On the other hand, at a small flank angle of 7°, little stock is left for finishing, and hence there is a tendency of the insert to slide on the material without proper cutting. The quality requirements of this kind elements are restrictive and, due to the specific design, in order to perform measurement of roughness on the thread surface, access to the examined surface is necessary, which involves its cutting, i.e., destruction. In connection with this, decisions concerning the meeting of the roughness bottom the original requirement is, in industrial practice, first made on the basis of visual assessment, and there is thus a need to undertake examinations confirming this subjective assessment.

## 2. Materials and Methods

The threads under examination were made on the Mori Seiki NL2500 machine. Semiproduct, in the form of a shaft of Inconel 718 material 340 mm long with an initially turned external diameter dimensioned 53 mm, was used; the diameter was also the major diameter of the threads. The threads were made in three kinds of outlines used in the aircraft industry: triangular—UNJ; trapezoidal symmetrical—ACME; and trapezoidal asymmetrical—BUTTRESS. Each was cut twice: first at the cutting speed of *v_c_* = 17 m/min and second at a speed of *v_c_* = 30 m/min (total of 6 threads). Each time, the thread was turned by a new insert being a nominal projection of the groove outline. The strategy of radial infeed with variable depth was applied to maintain a constant cross-section of the cut layer. Ecocool Global 10 cooling and lubricating liquid was used, with the concentration of 8% emulsion based on mineral oil and 92% water.

In the case of thread turning, the optimization possibilities are limited to changing the cutting speed, the number of passes (which determines the depth of cut) and the threading strategy. This study used a strategy that is dedicated to thread machining in Inconel type materials. Due to the high contact between the cutting edge and the workpiece, a large number of passes should be avoided, as the properties of this material cause rapid wear of the cutting edge. In the aerospace industry, the root radius has a tolerance of ±0.026 mm, so it is extremely important to maintain the original insert geometry by avoiding excessive wear. The cutting speed was adopted for testing on the basis of experience gained during production implementations on the main shafts of aircraft engines.

**Figure 1:**
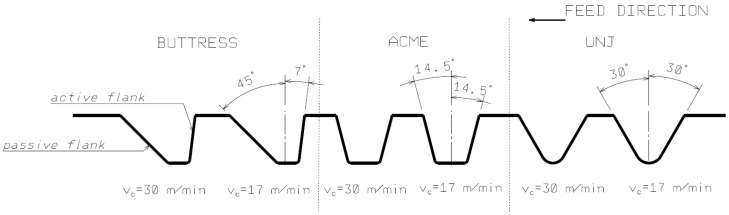
Schematic representation of the research object: trapezoidal asymmetrical thread—BUTTRESS; trapezoidal symmetrical thread—ACME; triangular thread—UNJ.

To facilitate the interpretation of the results, the following nomenclature for the individual surfaces has been adopted. The model is referred to as BUTTRESS thread, whose task it is to transfer loads in the direction of thrust with a smaller angle—in this case 7 degrees. The loaded flank is called the active flank; the unloaded one is called the passive flank. Similarly, for the other profiles, the side in the direction of the insert motion is called the passive one, the opposite side is called the active one. A schematic representation of the test object with the described angles of the flanks for each type of thread is shown in Figure 1.

**Figure 2:**
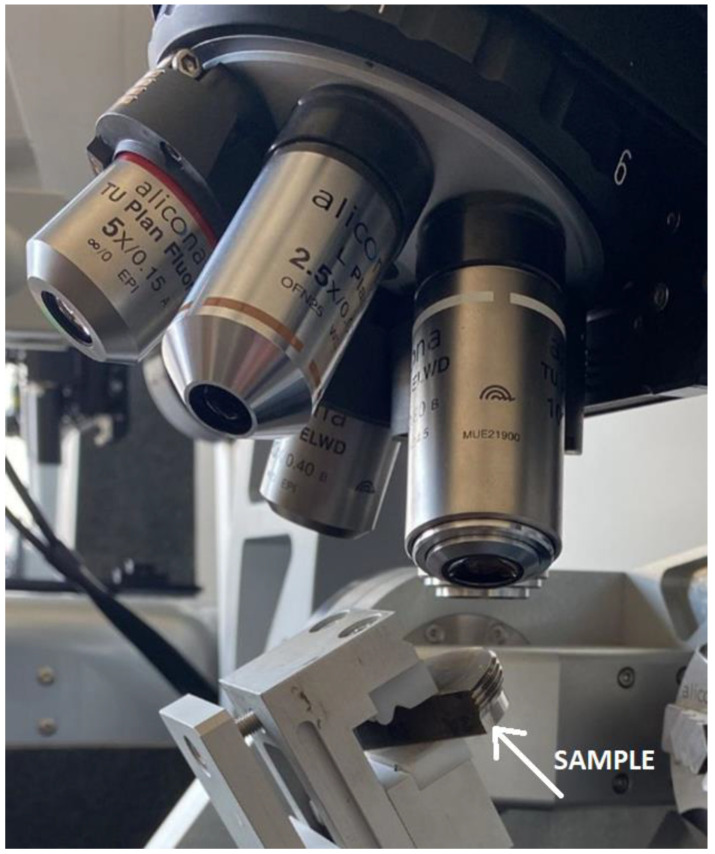
View of the measuring station with the InfiniteFocus G5 instrument.

The prepared threads were cut on an electro-erosion machine to provide access to the surfaces under assessment. A total of twelve side surfaces of the groove were examined (6 threads, two flanks each). The topography was assessed optically with the use of an InfiniteFocus G5 focus variation microscope made by Alicona company (Figure 2). Focus variation microscopy is a method of optical imaging of surfaces in which the microscope optical system performs a scanning motion above the surface, and the changes of the observed image sharpness are constantly recorded [25]. For the determination of the height coordinate of a surface point observed by the given pixel, the system uses the contrast information in relation to the neighbouring pixels. During the scanning motion, at predetermined intervals of the feed value (vertical resolution), information on the contrast value at a given point is recorded. After a full scanning motion is performed, a curve determining the coordinate of the maximum sharpness value is fitted to these values [26]. To obtain good measurement results, a procedure containing proper illumination was introduced [27].

The measurements were performed with the use of an optical configuration with a 50x magnification objective, allowing for roughness measurement with the arithmetical average of 0.05 µm. The cutout fragments of the threads were fixed in a miniature vice on a grip with a base, which allowed for the setting of the individual test pieces so that the measured surface could be arranged perpendicular to the optical axis. Each measurement area comprised the whole height of the active and passive sides. The other parameters of the measurement are shown in Table 1. The curvature of the shape of the surface under assessment was removed by means of a third-degree polynomial. A Gaussian filter with S = 2.5 µm and L = 0.25 mm was used. The spatial parameters of the topography, *Sa*, *Sq*, *Sku*, *Sp*, *Sv* and *Sz* were determined in accordance with the standard, ISO 25178-2 [28].

## 3. Results and Discussion

**Figure 3:**
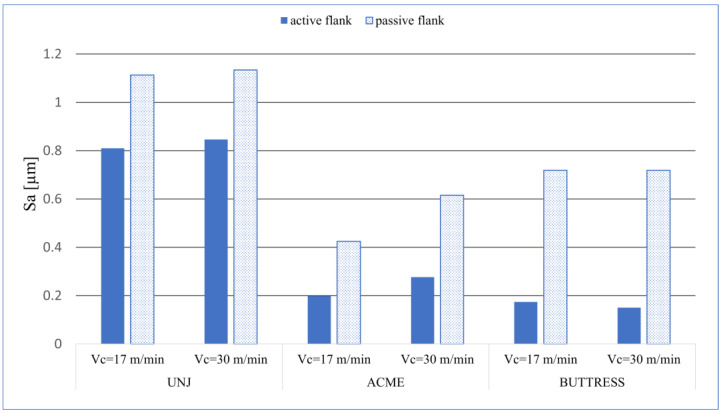
*Sa* values for the measured surfaces.

**Figure 4:**
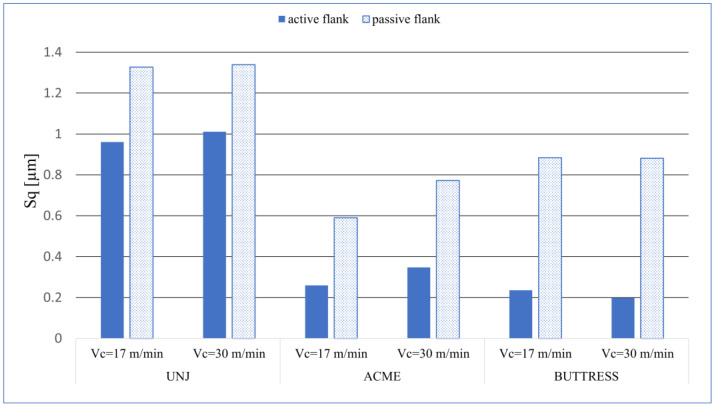
*Sq* values for the measured surfaces.

The measurement results of the selected topography parameters are shown in Table 2. For the sake of the analysis of the results, the values are presented in the form of graphs. Figure 3 shows the arithmetical mean height, *Sa*; Figure 4 shows the root-mean-square height of the surface, *Sq*.

**Figure 5:**
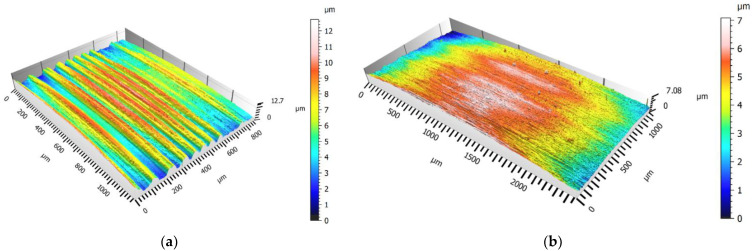
View of the surface topography: (**a**) UNJ thread, passive flank, *v_c_* = 30 m/min; (**b**) BUTTRESS, active flank, *v_c_* = 30 m/min.

Analysing the data shown in Figure 3 and Figure 4, we can see a correlation between them. This is of course natural, as these parameters are related to each other due to their mathematical description. On each thread, the obtained values of *Sa* and *Sq* on the passive side were higher than those on the active one. This may be a result arising from the direction of the chip flow and its difficult evacuation. Additionally, on this flank, greater cutting forces occurred due to the thrust of the insert. Regarding the influence of the cutting speed, no significant differences in the obtain results of *Sa* and *Sq* were detected. For the UNJ and ACME threads, the increase of the cutting speed resulted in a slight growth of the parameter values; for the BUTTRESS ones, this resulted in a slight drop. It is possible that the change *v_c_* from 17 m/min to 30 m/min was too small and, in the case of further increase, the differences would be more noticeable. It was noticed that, in the threads with triangular outline, the average values of the spatial roughness were higher than those in the trapezoidal threads. A maximum value of *Sa* = 1.134 µm (Figure 5a) was obtained for the passive flank of the UNJ thread and *v_c_* = 30 m/min and the minimum *Sa* = 0.151 µm (Figure 5b) for the active surface of the BUTTRESS thread, with *v_c_* = 30 m/min. The strategy of turning with the radial infeed without a shift in the axial direction in the subsequent passes might have influenced the obtained results. Despite the constant value of the cut layer being maintained, the allowances on the subsequent passes were much smaller, with smaller flank angles, and this is why the angle of 7° (BUTTRESS, active flank) appeared more “smoothed”.

The *Sa* and *Sq* parameters are often insufficient because they do not provide information concerning the distribution of the height of such features as vertices and pits, with the latter being important in respect to the matching of surfaces [28]. Much more detailed data concerning the surface characteristics can be determined by analysing the parameters, *Ssk* (surface asymmetry coefficient), i.e., skewness, and *Sku* (surface inclination coefficient), i.e., kurtosis. The first tells us whether the given surface has more pits (*Ssk* < 0) or protruding vertices (*Ssk* > 0). The *Sku* parameter shows the character of the irregularities and their distribution. A surface with a Gaussian height distribution has *Sku* value of 3. In the case of smaller values of *Sku* < 3, the surface under examination is flatter, and the occurring irregularities are mild. In the case of *Sku* > 3, the area contains uneven sharp peaks or pits. The higher the value of kurtosis is, the more defects there are on the surface. The coefficients are sometimes shown in the form of a *Sku–Ssk* map [30].

**Figure 6:**
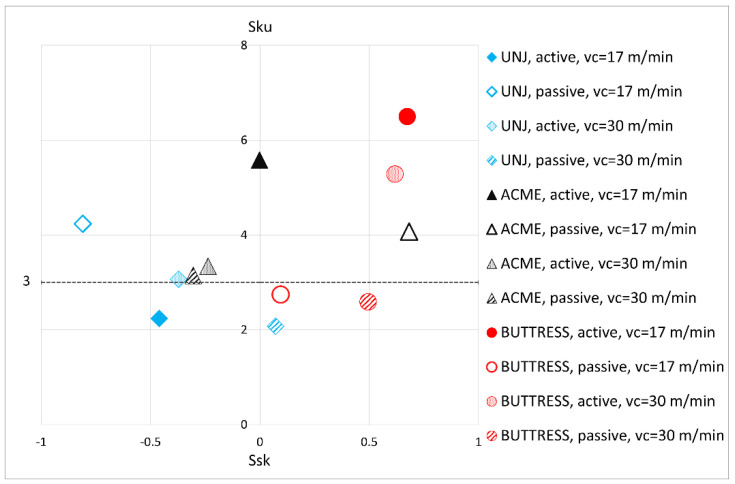
*Sku–Ssk* map for the measured surfaces.

In an analysis of the map containing data from the twelve examined surfaces (Figure 6), one does not see unambiguous relationships as were observed in the case of *Sa* and *Sq* parameters. Only four side surfaces of the thread groove (two of a triangular outline and two of a BUTTRESS thread) have the value of *Sku* < 3. It can be concluded that the unevenness on the thread surface are generally irregular and, as indicated by the *Ssk* values, sometimes pits prevail, sometimes peaks. The reason for the uneven distribution may be the large turning resistances which, consequently, influence the temperature increase in the cutting zone and increase the susceptibility to the occurrence of vibration. It is worth noticing that UNJ threads, despite higher *Sa* and *Sq* values, have a lower index of kurtosis than do the trapezoidal threads. This indicates that the irregularities on their surfaces are more predictable and milder. Considering that the *Ssk* parameter with mostly negative values or ones slightly above zero occurred for triangular threads, we can surmise that the defects on the UNJ surfaces are in most cases pits, which is advantageous in respect to the material abrasion. All four surfaces of the BUTTRESS thread are characterized by positive *Ssk* values (three of them >0.49), which indicates the prevalence of vertices and, in combination with the *Sku* values for the active sides well above 3, it can be concluded that the vertices are sharp. From the tribological point of view, the peaks are destroyed as a result of the decohesion of the top layer. In the case of the ACME type threads, it was observed that the pair of *Ssk–Sku* values had better results at a cutting speed of *v_c_* = 30 m/min compared to those at *v_c_* = 17 m/min.

**Figure 7:**
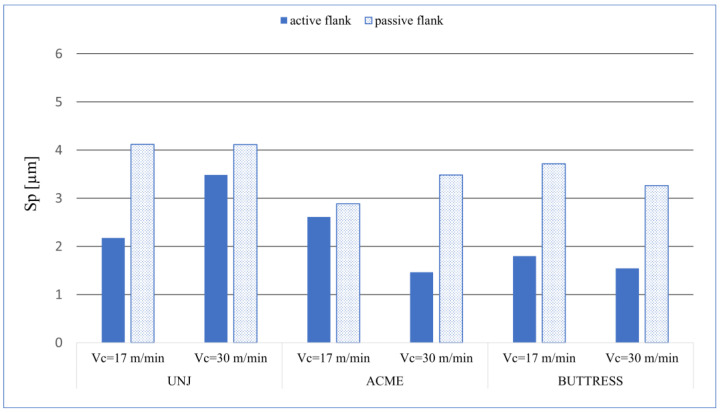
*Sp* values for the measured surfaces.

The last group of the analysed height and spatial parameters of roughness are as following: *Sp*—the maximum peak height; *Sv*—the maximum pit height; and *Sz* constituting the sum of the above, i.e., the distance from the lowest place to the highest one. In this manner, the parameters are mathematically connected. The application of the *Sp* parameter is related to the sliding surfaces. *Sv* can be applied to coated surfaces or ones requiring lubrication where pits or grooves are responsible for sticking fluids on the surface [31]. The results of *Sp* measurement are shown in a graph (Figure 7).

**Figure 8:**
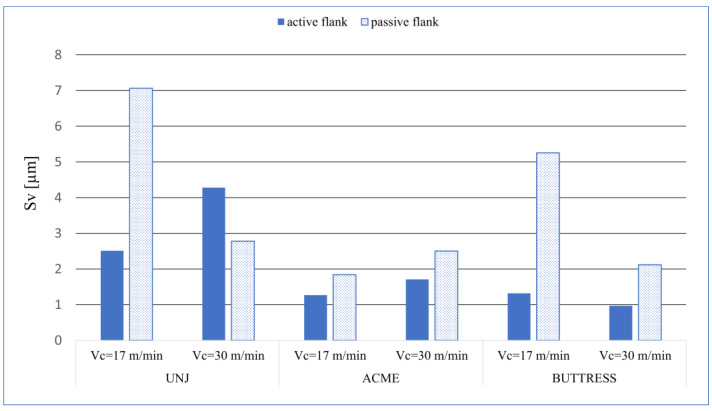
*Sv* values for the measured surfaces.

**Figure 9:**
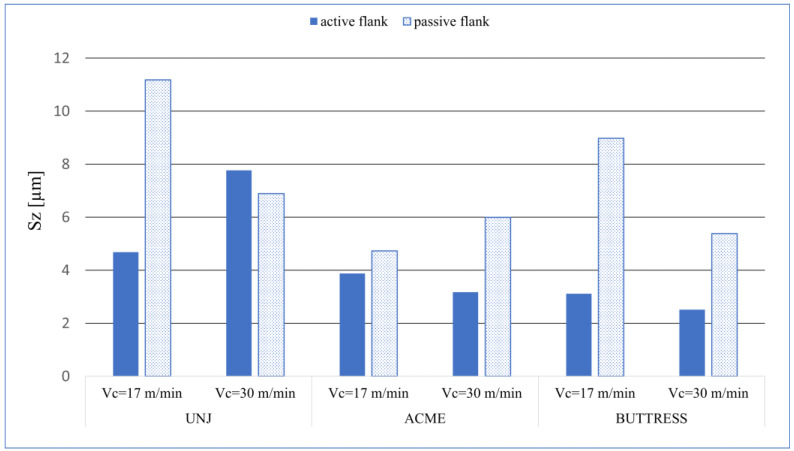
*Sz* values for the measured surfaces.

On each examined pitch, the passive surface had higher *Sp* values than did the active surface. A correlation of the values of *Sa* and *Sq* can be seen here. The maximum peak was measured on the passive flank of the triangular outline thread, and for both cutting speeds, *Sp* = 4.11 µm. Analysing the *Sv* values (Figure 8), we can see that the distribution of the value is less predictable as compared to *Sp*. On the three surfaces. the values differ from the other ones as follows: UNJ, passive side, *v_c_* = 17 m/min; UNJ, active side, *v_c_* = 17 m/min; and BUTTRESS, passive side, *v_c_* = 17 m/min, the. The maximum recorded pit occurred, as in the case of *Sp*, for the surface of the thread with a triangular outline, a passive side, and *v_c_* = 17 m/min, with *Sv* = 7.06. An anomaly was observed for this outline for the cutting speed of *v_c_* = 30 m/min where the active surface had higher *Sv* values than did the passive one. These results were confirmed by parameter *Sz* for which the mentioned surfaces also reached the highest values (Figure 9). The lowest values of *Sz* was obtained for the active sides of the BUTTRESS threads. From the *Ssk–Sku* map, we can see that the deformations on these surfaces are uneven and peaks, which is disadvantageous in respect to the prevailing wear. However, considering the values of height parameters, we can say that satisfactory sizes and character of the surface irregularities were obtained.

## 4. Conclusions

It can be stated that the analysis of the surface topography is certainly a complicated process and that a large number of the parameters obtained as result of examination may be misleading. Analysing the literature and referring to the publications contained in the reference list, one can notice that none of these works concern the study of the thread surface topography. It should be emphasized that the thread turning process, in addition to dealing with difficult-to-cut materials, is one of the most demanding in terms of mechanical processing. Moreover, it is worth noting that the examination of the surface topography using optical methods should be carried out perpendicularly to the assessed surface. In the case of threads, this surface is not directly accessible, and in order to measure the topography, the element must be destroyed by cutting, which certainly makes this test less common.

It is difficult to quantify the effect of the parameters *Sa*, *Sq*, *Sp*, *Sv*, *Sz*, *Ssk* and *Sku* on the quality of machined parts, but there is an increasing number of studies reporting the the 3D roughness parameters used to evaluate the machined surface. Papers published by Pawlus et al. [28], Krolczyk et al. [3] and Kacalak et al. [32] concern the use of the above-mentioned parameters to assess the condition of the surface. The numerous studies presented in these works do not show to what extent individual parameters affect functionality, but rather the phenomena related to the functionality they influence. According to these studies, the parameters *Sa*, *Sq*, *Sp*, *Sv* and *Sz* have an influence on the surface contact, lubrication, friction, wear and fatigue, while the parameters *Ssk* and *Sku* have an influence on the surface contact, friction and wear. It is known which parameters are responsible for which performance characteristics, but it is indeed impossible to determine the magnitude of this influence on this basis. The aim of this study was to investigate the influence of factors such as cutting speed, feed direction and flank angle, which differ across the individual thread types, on the obtained values of the parameters *Sa*, *Sq*, *Sp*, *Sv*, *Sz*, *Ssk* and *Sku*. In industrial practice, in principle, topography assessment is not used at present, and this area is still being developed. At the moment, surface quality is assessed using the *Ra* parameter. However, given the design of the thread and the tasks these elements fulfil, the determination of the topography allows for a more accurate understanding of the surface texture. Taking into account the fact that thread topography studies (especially when it comes to the material Inconel 718) are generally not performed, as confirmed by the literature review, we concluded that this publication provides an interesting basis for delving into the topic of assessing the flank surfaces of threads machined during the turning process. We are in full agreement that this work only shows the obtained values of the individual parameters, with their comparison taking into account the previously mentioned factors. We were not able to quantify the influence of individual parameters on the quality of manufactured components, and further research in this direction should be considered. We are aware that if the functionality of a manufactured component is to be assessed well on the basis of determined topography parameters, several parameters have to be considered simultaneously, which poses an additional difficulty.

Based on our experience in the field of machining, we have indicated in this paper several potential reasons for the obtained results; however, the mechanism has not been investigated in this study. Various types of threads were tested where particular types differed primarily in the angles of the flanks, which had a significant impact on the distribution of cutting forces during turning. With large BUTTRESS thread angles, i.e., 45°, there are large forces in the radial direction, which may result in an increased tendency to vibration, especially in low-rigid elements. On the other hand, at a small flank angle of 7°, little stock is left for finishing, and hence there is a tendency of the insert to slide on the material without proper cutting. In order to confirm the reasons speculated, the need for further research is noted. Certainly, tests should be carried out on a larger number of components and a wider range of varying cutting parameters, e.g., cutting speeds. An interesting development of the research conducted here could be placing a vibration sensor and examining vibrations during the turning of individual threads. Nevertheless, it should be pointed out that similar relationships have been described by other researchers. Gunay indicated that the increasing of radial force associated with the infeed strategy could causes poor surface quality [33]. He at al. proved the nonlinear effect of cutting speed on thread surface quality when turning Inconel 718 [24]. An at al. shown that flank wear on the left edge is more serious than that of the right edge during the BUTTRESS thread turning process [34]. This may explain why the passive side had a worse surface quality than did the active side. In the same work, researchers concluded that irregular chip may scrape the machined surface and worsen the surface quality. Jiang et al. claimed that vibrations and tool wear are caused by many factors, making it difficult to identify the influencing aspects of turning stability and to control the process of the turning thread [35].

The present work represents an analysis of the topography of the thread surface, which brings additional difficulties due to the complicated shape of the surface and large loads during machining, causing uneven irregularities. Nevertheless, a number of relationships have been noticed:

- On the surfaces cut in the direction of the turning, worse quality was obtained, in contrast to the opposite surfaces, which may be a result of the problems of chip evacuation and the distribution of the cutting forces which in turn leads to greater wear of the left side of cutting edge. This result suggests that the selection of the cutting direction when making decisions concerning the strategy of turning external threads is of great importance. This is important from the point of view of quality requirements, where the thread side loaded during work of a thread joint has lower roughness values, resulting from the increased susceptibility to abrasion.
- The side surfaces of the threads with triangular outline had worse surface quality than did those of the trapezoidal threads in terms of the average *Sa* and *Sq* parameters. This may be an effect of the adopted turning strategy with the radial infeed where, with smaller flank angles, lower allowances arise, which, consequently, results in additional “smoothing” of that surface.
- Analysis of the *Sku–Ssk* map (Figure 5) evidences the presence of many uneven irregularities on the machined surfaces. Considering the *Sku* and *Ssk* values, the UNJ threads have a more predictable distribution of ups and downs. In addition, pits predominate, what is advantage in respect to wear.
- Extremely high values of *Sp*, *Sv* and *Sz* occurred on the passive sides, which is related to the values of *Sa* and *Sq*. This should be taken into consideration when manufacturing threads which will be subjected to loads due to the susceptibility to larger wear of the surface containing sharp vertices.
- As regards the comparison of the two cutting speeds, in most cases lower values of the height parameters of the topography were obtained for *v_c_* = 17 m/min, but the differences were not significant. The roughness parameters for the UNJ and ACME threads were smaller for the cutting speed *v_c_* = 17 m/min, while for the BUTTRESS threads, these parameters were smaller for *v_c_* = 30 m/min. This fact does not confirm the conclusions made by Kümmel [36], who found that increasing the cutting speed had a positive effect on the surface roughness. It is worth noting, however, that visual assessment suggested that the surface turned at a higher cutting speed is smoother. This is a very important conclusion and informs us that the assessment is, to a large extent, subjective, and one should be extremely careful when making decisions regarding the acceptance of meeting the quality criteria of the manufactured product based merely on the visual assessment.
- Similar to Krolczyk et al. [6], we focused our study on the assessment of roughness parameters describing the condition of the surface layer. The condition of the cutting inserts after the performed tests is not mentioned in this paper; however, we observed built-up edge (BUE) on the rake face on the cutting inserts used during machining with a lower cutting speed. It is difficult to determine the effect of built-up edge on the roughness of the machined surfaces, as it is ambiguous.

## Figures and Tables

**Table 1:** Topography measurement parameters with the InfiniteFocus G5 device.

Lens	Vertical Resolution	Horizontal Resolution	Measurement Time	Lighting	Measure Area
50x	0.041 μm	2 μm	~16 min	Coaxial	~1.2 mm × 0.9 mm

**Table 2:** Topography measurements results according to ISO 25178-2 [29].

Thread Type	*V_c_* [m/min]	Flank	*Sa* [µm]	*Sq* [µm]	*Ssk*	*Sku*	*Sp* [µm]	*Sv* [µm]	*Sz* [µm]
**UNJ**	**17**	**Active**	0.810	0.960	−0.460	2.238	2.176	2.511	4.687
		**Passive**	1.113	1.327	−0.811	4.237	4.119	7.064	11.183
	**30**	**Active**	0.846	1.011	−0.372	3.068	3.485	4.282	7.767
		**Passive**	1.134	1.338	0.072	2.079	4.114	2.781	6.895
**ACME**	**17**	**Active**	0.199	0.259	−0.002	5.581	2.611	1.272	3.883
		**Passive**	0.425	0.590	0.683	4.072	2.888	1.842	4.729
	**30**	**Active**	0.277	0.348	−0.237	3.340	1.466	1.712	3.178
		**Passive**	0.615	0.772	−0.305	3.148	3.485	2.506	5.990
**BUTTRESS**	**17**	**Active**	0.174	0.235	0.674	6.498	1.800	1.318	3.118
		**Passive**	0.719	0.884	0.095	2.742	3.719	5.256	8.975
	**30**	**Active**	0.151	0.198	0.618	5.283	1.547	0.970	2.517
		**Passive**	0.718	0.881	0.495	2.590	3.263	2.121	5.384

## Data Availability

Data sharing is not applicable to this article.
